# Phylogeny of Maleae (Rosaceae) Based on Multiple Chloroplast Regions: Implications to Genera Circumscription

**DOI:** 10.1155/2018/7627191

**Published:** 2018-03-19

**Authors:** Jiahui Sun, Shuo Shi, Jinlu Li, Jing Yu, Ling Wang, Xueying Yang, Ling Guo, Shiliang Zhou

**Affiliations:** ^1^State Key Laboratory of Systematic and Evolutionary Botany, Institute of Botany, Chinese Academy of Sciences, Beijing 100093, China; ^2^University of the Chinese Academy of Sciences, Beijing 100043, China; ^3^College of Life Science, Hebei Normal University, Shijiazhuang 050024, China; ^4^The Department of Landscape Architecture, Northeast Forestry University, Harbin 150040, China; ^5^Key Laboratory of Forensic Genetics, Institute of Forensic Science, Ministry of Public Security, Beijing 100038, China; ^6^Beijing Botanical Garden, Beijing 100093, China

## Abstract

Maleae consists of economically and ecologically important plants. However, there are considerable disputes on generic circumscription due to the lack of a reliable phylogeny at generic level. In this study, molecular phylogeny of 35 generally accepted genera in Maleae is established using 15 chloroplast regions.* Gillenia* is the most basal clade of Maleae, followed by* Kageneckia* +* Lindleya*,* Vauquelinia*, and a typical radiation clade, the core Maleae, suggesting that the proposal of four subtribes is reasonable. In the core Maleae including 31 genera, chloroplast gene data support that the four* Malus*-related genera should better be merged into one genus and the six* Sorbus*-related genera would be classified into two genera, whereas all* Photinia*-related genera should be accepted as distinct genera. Although the phylogenetic relationships among the genera in Maleae are much clearer than before, it is still premature to make a formal taxonomic treatment for these genera.

## 1. Introduction

Rosaceae or rose family, consisting of approximately 4,828 species in 91 genera [1], is of great economic and ecological importance. Many species are cultivated for their fruits or as ornamentals. The monophyly of the family is implied by the presence of unique floral structures and strongly supported by* rbcL* phylogeny [2]. However, the rose family displays a considerable diversity in morphology and anatomy, and it had been generally subdivided into four subfamilies, that is, Rosoideae, Spiraeoideae, Amygdaloideae (incorrectly Prunoideae), and Maloideae. Such a subdivision was recently challenged by molecular phylogenies of* matK* and* trnL-F* [3], six nuclear and four chloroplast regions [4], and hundreds of nuclear genes [5]. A formal three-subfamily classification was proposed: Dryadoideae, Rosoideae, and Amygdaloideae (incorrectly Spiraeoideae in [4]). Dryadoideae was separated from Rosoideae, and Spiraeoideae and Maloideae were merged with Amygdaloideae (incorrectly Spiraeoideae).

One of the most striking changes in the new classification is that traditional Maloideae became subtribe Malinae (incorrectly Pyrinae). Here we use tribe Maleae instead of supertribe Pyrodae sensu [4] to include the traditional pome-bearing Maloideae plus* Gillenia* Moench (=*Porteranthus* Britton),* Kageneckia* Ruiz & Pav.,* Lindleya* Kunth, and* Vauquelinia* Corrêa ex Bonpl. in traditional Spiraeoideae [4, 6–9].

Maleae consists of about 1,000 species, occurring mostly in the temperate Northern Hemisphere. The tribe includes many well-known fruit crops such as apple (*Malus pumila* Mill.), pear* (Pyrus spp*.), loquat (*Eriobotrya japonica* (Thunb.) Lindl.), and black chokeberry (*Aronia melanocarpa* (Michx.) Elliott), as well as many ornamentals. The core Maleae is characterized by a synapomorphic pome, a type of accessory fruit that does not occur in other Rosaceae plants [10], and a basal chromosome number, *x* = 17. With the addition of* Gillenia*,* Kageneckia*,* Lindleya*, and* Vauquelinia*, the tribe has drupaceous or follicle fruits and *x* = 9 (or 15) as well [2, 4, 6, 9, 11–13].

The origin of core Maleae (*x* = 17) has long been considered an example of allopolyploidization between the species with *x* = 9 in traditional Spiraeoideae and the species with *x* = 8 in traditional Amygdaloideae [14–17]. However, recently discovered genomic data suggested an origin via autopolyploidization followed by aneuploidization around 50 million years ago [18, 19]. In contrast, the allopolyploid nature of core Maleae was confirmed by GBSSI, which had four copies in the core Maleae but only two copies in other groups [6].

Polyploidization affects systematics at both generic and specific levels. It is unlikely to resolve the polytomy of ancestral populations that have just a few closely related species involved in historical speciation and subsequent diversification, owing to the lack of phylogenetically informative signals and incomplete lineage sorting. The merging of several genomes into one species enriches the pool of available genetic combinations and the survival of recombinants is overcome by apomixis. Maleae is one such species-rich tribe of genera but is difficult to classify.

The pome-bearing plants have been generally subdivided into two groups: one with connate endocarps and the other with polypyrenous drupes [10, 20–22]. However, such subdivisions of the core Maleae do not receive support from molecular data. Considerable controversies exist on circumscriptions of genera based on morphology or anatomy [21, 23–37]. * Sorbus* L. is considered to include both the pinnate-leaved species (*Sorbus s.s.* and* Cormus *Spach) and the simple-leaved species (*Aria* (Pers.) Host,* Micromeles* Decne.,* Chamaemespilus* Medik., and* Torminalis* Medik.) [21, 34, 38, 39].* Aronia *Medik.,* Heteromeles* M. Roem.,* Pourthiaea* Decne., and* Stranvaesia *Lindl. are either merged together with* Photinia* Lindl. or accepted as distinct genera [37, 40, 41]. Varying opinions are also found among* Malus* Mill.,* Docyniopsis* Koidz., and* Eriolobus* (DC.) M. Roem., as well as among* Pseudocydonia *(C. K. Schneid.) C. K. Schneid.,* Cydonia* Mill., and* Chaenomeles* Lindl. [22, 27, 37]. Lack of consensus treatment among these genera is a result of uncertainty about genetic relationships among all entities (or genera in the narrow sense) and molecular data have been used to reveal their phylogenies. Both chloroplast DNA sequences [2, 4, 7, 9, 42] and nuclear DNA sequences ([4, 6–8, 12, 43]; Lo et al., 2012) have been tried, and it is clear that the monophyly of each entity is highly supported; the major unsolved problem is the phylogeny among entities. To elucidate the intricate relationships among these entities (the generally recognized genera in the narrow sense within Maleae), we sampled representative species for each of them, including the occasionally accepted genera and subgenera, and constructed their phylogeny using 15 chloroplast regions. Our results are expected to be helpful for the correct circumscription of genera in Maleae.

## 2. Materials and Methods

### 2.1. Taxon Sampling

A total of 41 species were examined: 35 species representing all generally recognized genera of Maleae and five species representing other major lineages of Amygdaloideae and* Rosa rugosa* Thunb. as an outgroup (Table 1). All samples were collected under appropriate permits: Herbarium, Institute of Botany, CAS; Beijing Botanical Garden, CAS; Kunming Botanical Garden, CAS; and Xishuangbanna Tropical Botanical Garden, CAS; and other places listed in Table 1.

### 2.2. DNA Extraction, Amplification, and Sequencing

Total genomic DNA was extracted using the mCTAB method [44] and purified using the Wizard DNA Clean-Up System (A7280, Promega Corporation, Madison, WI). Fifteen chloroplast regions, that is,* atpB-rbcL*,* matK*,* ndhF*,* petA-psbJ*,* psbA-trnH*,* psbM-trnD*,* rbcL*,* rpl16*,* rpl20-rps12*,* rps16*,* trnC-ycf6*,* trnH-rpl2*,* trnL-trnF*,* trnS-trnG*, and* ycf1*, were used in this study. Fourteen primer pairs published in Dong et al. [45, 46] were synthesized by Sangon Biotech (Shanghai) Co. Ltd. (Beijing, China) and used to amplify and sequence these regions (Table 2). Sequences of* rps16* were downloaded from GenBank. The polymerase chain reaction (PCR) amplifications were carried out in a mixture volume of 20 *μ*L, containing 1x Taq buffer (1 mol/L KCl; 20 mmol Tris-HCl pH 9.0; 1% Triton X-100), 2.0 *μ*L dNTPs (2 mmol/L), 1.0 *μ*L each of the primers (5 *μ*mol/L), 20 ng of genomic DNA, and 1 unit of Taq polymerase. PCR was conducted using a C1000 Thermal Cycler (Bio-Rad Laboratories, Hercules, CA, USA). The program started at 94°C for 3 min, followed by 35 cycles at 94°C for 30 s, 50°C for 30 s, and 72°C for 2 min, and ended at 72°C for 5 min. The PCR products were purified with an equal mixture of 40% PEG 8000 and 5 mol/L NaCl, followed by a washing step with 80% ethanol. The PCR products were then Sanger sequenced for both strands on an ABI 3730xl DNA Analyzer using BigDye Terminator cycle sequencing kit v3.1 (Applied Biosystems, Foster City, CAS, USA) following the manufacturer's instructions.

### 2.3. Sequence Data Preparation and Evaluation

The newly generated sequences were checked and assembled using Sequencer 4.7 (Gene codes Corporation, Ann Arbor, Michigan, USA). The resulting sequences (Supplementary Table S2) were combined with the published sequences of Maleae species downloaded from GenBank (Supplementary Table S1), aligned with Clustal X [47, 48], and then manually adjusted with Se-Al 2.0 [49]. Each individual gene dataset was then subjected to several rounds of phylogenetic evaluations to select reliable sequences. Sequences that were misidentified or exhibited large amounts of missing data were excluded from the datasets. To predict the phylogenetic performance of individual gene partitions, the variability of genes was parameterized by DnaSP 5.0 [50] and PAUP 4.0b10 [51] using nucleotide diversity, the number of polymorphic sites, and so forth. Prior to concatenating the dataset of each marker, incongruence length difference (ILD) tests were performed on all 15 datasets. The datasets were finally concatenated into one final data matrix using SequenceMatrix [52] for phylogenetic analyses.

### 2.4. Phylogenetic Analysis

Phylogenetic analyses were performed on single gene datasets and the concatenated dataset, using PAUP 4.0b10 [51] for maximum parsimony (MP), RAxML v.8.1.24 [53] for maximum likelihood (ML), and MrBayes 3.2.2 [54] for Bayesian inference (BI). The MP analysis used a heuristic search that treats all characters as equally weighted and unordered, obtaining the starting trees by stepwise addition, random stepwise addition of 100 replicates, TBR, and MulTrees enabled. Branch support for MP trees was assessed with 1,000 bootstrap replicates and all trees were saved at each replicate. ML analysis was performed with 1,000 nonparametric bootstrap replicates (BP). The suitable substitution model (GTR + I + G) for BI analysis was inferred by using ModelTest version 3.7 [55] under the Akaike information criterion. Default settings were used for the MrBayes run, and 2 × four chains were run for ten million generations, sampled every 1,000 generations. Posterior probabilities (PP) were calculated from the majority consensus of all of the sampled trees when the standard deviation of the split frequencies (SDSF) permanently fell below 0.01, and the trees sampled during the burn-in phase were discarded.

## 3. Results

### 3.1. Sequence Variability within Maleae

The ILD tests did not suggest serious conflicts between datasets of the 15 chloroplast regions (*p* = 0.11). The variability of the 15 examined DNA regions within Maleae are given in Table 3, together with the MP tree scores of all taxa. The longest DNA region was* matK *(La = 2045), and the shortest was* trnH-rpl2 *(La = 205). Of the 15 chloroplast regions,* psbA-trnH *was the most variable fragment with a *π* (nucleotide diversity) value of 0.02547, whereas* trnH-rpl2* was the least variable fragment with a *π* value of 0.00391. The* psbA-trnH*,* petA-psbJ*,* ycf1*,* trnS-trnG*, and* trnC-ycf6* gene regions were more variable (*π* > 0.010) than other genes.

The concatenated data matrix of the 41 taxa reached an aligned length of 17,554 bp with 1,266 parsimony-informative characters. MP searches yielded one best tree with a consistency index (CI) of 0.779, a retention index (RI) of 0.637, and a tree length of 5,974.

### 3.2. Phylogenetic Relationships of the Basal Maleae

**Figure 1:**
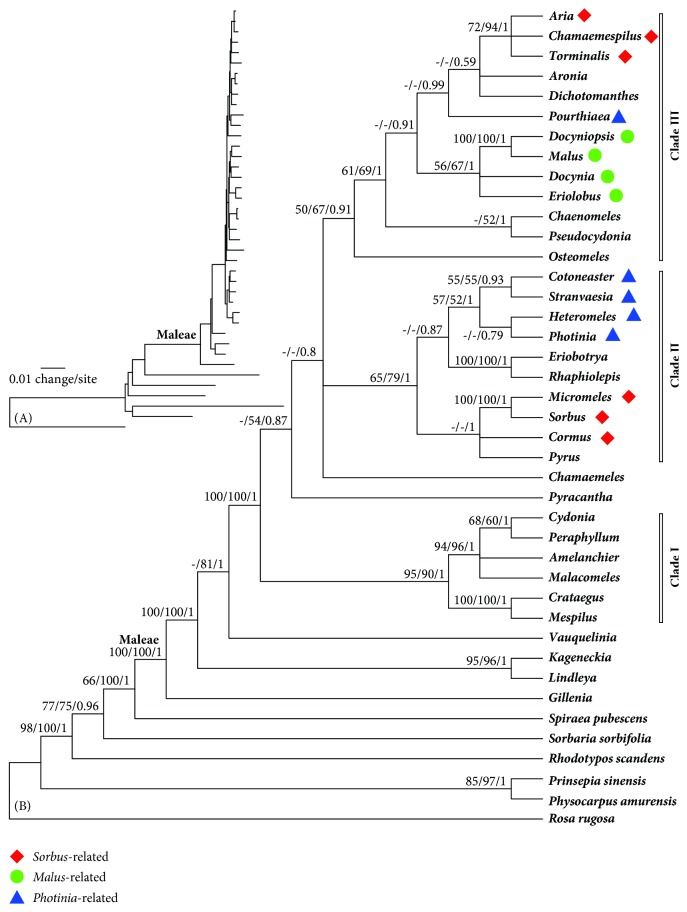
Phylogenetic relationships of Maleae based on a concatenated dataset of 15 chloroplast gene regions. (A) Bayesian phylogram with branch lengths. (B) 50% majority-rule Bayesian consensus tree with bootstrap supports. Values beside the branches are the bootstrap percentages from maximum parsimony analysis, maximum likelihood analysis, and Bayesian posterior probabilities. “-” indicates a branch collapse in the maximum parsimony and maximum likelihood trees.

Polytomies were observed in all the 15 best trees based on each chloroplast region. However, the tree topologies were similar; we thus concatenated them to build best resolved phylogenetic trees using MP, ML, and BI methods. The consensus trees from the MP, ML, and BI analyses showed substantially identical topologies and the monophyly of Maleae was recovered (Figure 1). A long branch leads to the crown taxa of Maleae, including* Gillenia*,* Lindleya*,* Kageneckia*, and* Vauquelinia* (Figure 1(A)), and this branch is strongly supported (Figure 1(B)) (PB = 100, BP = 100, PP = 1).

An earliest and remarkable divergence of* Gillenia* is clearly indicated in Figure 1.* Lindleya* and* Kageneckia* form a monophyletic clade and they diverged slightly earlier than* Vauquelinia*. The divergence among* Lindleya* +* Kageneckia*,* Vauquelinia,* and the core Maleae happened within a very short span of time, as indicated by the very short branches. All the three branches are well supported. The basal branching pattern of Maleae may serve as the foundation of subtribal division within the tribe if necessary.

### 3.3. Phylogenetic Relationships within the Core Maleae

The monophyly of the core Maleae is clearly indicated (Figure 1(A)) and well supported (Figure 1(B)). The genera in the core Maleae seem to be from the second radiation event. Nevertheless, three multigenus clades within the core Maleae are recognizable. Clade I consists of* Amelanchier* Medik.,* Crataegus* L.,* Cydonia* Mill.,* Malacomeles* (Decne.) Decne.,* Mespilus* L., and* Peraphyllum* Nutt. and is well supported (PB = 95, BP = 90, PP = 1). A very close relationship between* Crataegus* and* Mespilus* is indicated. Clade II (PB = 65, BP = 79, PP = 1) consists of four* Photinia*-related, three* Sorbus*-related, and three distinct entities. There are four pairs of genera in this clade but only two of them are highly supported (PB = 100, BP = 100, PP = 1), namely,* Eriobotrya* Lindl. versus* Rhaphiolepis *Lindl. and* Micromeles* Decne. versus* Sorbus* L.* Stranvaesia *Lindl. was closer to* Cotoneaster* Mecik. rather than to* Photinia *Lindl. Clade III consists of four* Malus*-related, one* Photinia*-related, three* Sorbus*-related, and five distinct entities and is weakly supported (PB = 50, BP = 67, PP = 0.91). Very close relationships were revealed between* Malus*-related* Docyniopsis *Koidz. and* Malus* Mill. (PB = 100, BP = 100, PP = 1) and among the* Sorbus*-related* Aria *(Pers.) Host,* Chamaemespilus *Medik., and* Torminalis *Medik. (PB = 72, BP = 94, PP = 1).* Docynia* Decne. and* Eriolobus* (DC.) Roem. also fall in the same clade as* Docyniopsis *and* Malus*, but the clade is only weakly supported (PB = 56, BP = 67, PP = 1).* Chaenomeles* Lindl. is related to* Pseudocydonia* (C. K. Schneid.) C. K. Schneid. (BP = 52, PP = 1).

## 4. Discussion

### 4.1. Taxa and Gene Sampling Strategies

It is often a challenge to reconstruct the phylogeny of taxa from recent radiation events due to unclear relationships among subdivisions and low resolution of markers. Maleae is such a taxon and that is why early studies failed to establish solid phylogenetic relationships among its subdivisions. In “A checklist of the subfamily Maloideae (Rosaceae)” [21], only 23 genera were accepted. Six* Sorbus*-related entities, that is,* Aria*,* Chamaemespilus*,* Cormus*,* Micromeles*,* Sorbus*, and* Torminalis*, were proposed to be merged. To test the distinctness of these taxa, we included all of them in this study.* Malus*-related and* Photinia*-related entities have similar taxonomic problems. Inclusion of representative species from other lineages of Amygdaloideae was to test the monophyly of Maleae. Considering that the monophyly of the genera in the narrowest sense has been well verified, 35 entities with one representative species each were sampled for being the most inclusive and economically affordable.

Sampling chloroplast regions as molecular markers for Maleae is another challenge. The tribe was found to be quite young and the core Maleae was even younger. Chloroplast markers have showed very low resolutions in previous studies [2, 4, 7, 9, 42]. Therefore, the most variable regions suggested by Dong et al. [56] were used together with the four conventional DNA barcodes,* matK*,* psbA-trnH*,* rbcL*, and* ycf1* [46]. By doing so, the major groups in Maleae were well resolved.

### 4.2. Phylogenetic Relationships among Major Groups of Maleae

The phylogeny (Figure 1) strongly suggests inclusion of* Gillenia*,* Kageneckia*,* Lindleya*, and* Vauquelinia* in Maleae (incorrectly Pyrodae in [7] and in [4]).* Gillenia* seemed to diverge slightly early, and* Kageneckia *+* Lindleya*,* Vauquelinia*, and the core Maleae are quite probably from the first radiation event. Although the inclusion of* Kageneckia*,* Lindleya*, and* Vauquelinia* into Maleae seems disagreeable with respect to fruit types, their basal chromosome number (*x* = 15 or 17) suggests that they had probably experienced similar speciation events. They bridge the gap between the core Maleae and true spiraeoid* Gillenia*. The inclusion of* Gillenia* in Maleae verifies that the core Maleae is from spiraeoid members within the tribe or other tribes in Amygdaloideae. Unfortunately, transcriptome data of nuclear genes did not provide more information for the issue of origin of the core Maleae because Figure 1 is substantially similar to the tree topologies based on transcriptome data in [5]. Given that* Gillenia* is the only diploid member in Maleae, the ancestor of* Gillenia* or its close relatives must be the maternal parent of the extant Maleae.

### 4.3. Phylogenetic Relationships within the Core Maleae

The core Maleae is a natural group with several synapomorphic characters, such as syncarpous ovaries, epigynous flowers, and fleshy fruits derived from the hypanthial ovary [57–59]. It could be better classified as subtribe Malinae (incorrectly Pyrinae) if necessary. The Malinae diverged into genera by radiation which is the second radiation event in Malese. The genera are so closely related that it is unnecessary to subdivide this subtribe further.

#### 4.3.1. Generic Pairs and Their Taxonomic Status

There are four generic pairs, for which the taxonomic status needs to be clarified. Firstly, a close genetic relationship between* Crataegus* and* Mespilus* has been revealed by Lo and Donoghue [9] in detail, which is confirmed here again. If* Mespilus* were to be accepted as a distinct genus,* Crataegus* would become a paraphyletic group. Although* Mespilus* was merged with* Crataegus*, its distinction was still recognized by giving it the taxonomic rank of section in* Crataegus *[9]. Secondly, reduction of* Pseudocydonia* to* Chaenomeles* was done by Gu and Spongberg [60], and their treatment is supported by Campbell et al. [7]. However, their close relationship does not receive high support in this study (BP = 52, PP = 1). They have diverged for some time because the clade does not show remarkable branch length. They had better be considered distinct genera. Thirdly,* Stranvaesia* is morphologically similar to both* Photinia* and* Cotoneaster* and sometimes submerged into* Photinia* [61]. Phylogeny based on chloroplast regions done by Campbell et al. [7] indicated a close relationship between* Stranvaesia* and* Pyracantha*. However, our data support a close relationship between* Stranvaesia* and* Cotoneaster*, a relationship also supported by GBSSI-1B [7]. Fourthly, although very close morphological and genetic relationships have been revealed in almost all studies involving* Eriobotrya* and* Rhaphiolepis*, there has been no suggestion yet to merge them into one, despite the existence of their hybrids [62]. Genetic divergence between them is very recently because their clade has a relatively long branch.

#### 4.3.2. Sorbus-Related Genera

So far, no well-sampled phylogeny of all* Sorbus*-related taxa is available, owing to the genetic complexity of the group and difficulties in sampling. There are ca. 250 species belonging to mainly temperate areas in the Northern Hemisphere [21], and six narrowly defined genera or subgenera under* Sorbus* in the broadest sense are usually accepted:* Aria*,* Chamaemespilus*,* Cormus*,* Micromeles*,* Sorbus*, and* Torminalis* [21, 37–39, 43, 58, 63–72]. These taxa fall into two clades in this study (Figure 1).* Sorbus sensu stricto* and* Cormus* with compound leaves nested in Clade I, and* Aria*,* Chamaemespilus*, and* Torminalis* with lobed or unlobed simple leaves nested in Clade III.* Micromeles*, a genus created to contain the species without persistent calyx lobes, is not a natural entity and is, therefore, unacceptable [8].* Micromeles folgneri* in this study is a synonym of* Sorbus folgneri*. Although* Cormus* had been considered a synonym of* Sorbus*, its taxonomic position remains to be determined.* Aria*,* Chamaemespilus*, and* Torminalis* in Clade III are closely related and they had better merge into one separate from* Sorbus*.

*Sorbus*-related entities are notorious in taxonomy due to complexity in phenotypes resulting from interspecific hybridization and facultative agamospermy [39, 69, 70]. Apomictic microspecies confound systematic resolution of agamic complexes using nuclear markers [71]. Phylogenies based on nuclear genes may suffer from paralogue problems and chloroplast markers would work better at the very beginning when no clear ideas are available for classification.

#### 4.3.3. Photinia-Related Genera

Four genera in their narrowest sense,* Aronia*,* Heteromeles, Pourthiaea*, and* Stranvaesia*, are considered related to* Photinia* [24, 25, 29, 31, 37]. This study demonstrates that all the five genera are superficially similar but actually distinct groups.* Heteromeles* possesses a soft pyrene, while* Photinia* possesses a soft core [16, 37, 58].* Stranvaesia* is distinguishable from* Photinia* by the dehiscent fruits at maturity. Although Guo et al. [73] stated with quite certainty that* Stranvaesia* must be merged into* Photinia*, we believe that further studies are needed for a reliable conclusion.* Pourthiaea* belongs to Clade III instead of Clade II as other genera. It is readily separable from other genera by characters such as deciduous habit, warty peduncles and pedicels, and a pulp structure in the fruits [41]. The distinction of* Pourthiaea* is further supported by leaf epidermis and wood anatomy [74, 75].* Aronia* is not a sister group of* Pourthiaea* because the clade is poorly supported, a conclusion that is similar to the earlier studies ([8, 9, 73]).

#### 4.3.4. Malus-Related Genera

The four genera,* Docynia*,* Docyniopsis*,* Eriolobus*, and* Malus*, really have very close relationships among them, especially between* Docyniopsis* and* Malus*. Some species have been transferred among the four genera by different authors. The monophyly of a clade comprising the four genera is indicated by our chloroplast data (Figure 1(B)). There are only two species in* Docynia*, two species in* Docyniopsis*, and one species in* Eriolobus*. Considering that these species are nested within* Malus* [18], these genera could better be merged with* Malus*.

## 5. Conclusions

This molecular phylogenetic study was conducted on all the genera of Maleae, by using 15 variable chloroplast gene regions. Four major clades are well resolved and the branches are well supported. These clades could be classified into four subtribes. The first radiation event gave birth to* Lindleya* +* Kageneckia*,* Vauquelinia,* and the ancestor of the core Maleae, and the second radiation event triggered the divergence of the genera in the core Maleae within a short time period. Within the core Maleae the four* Malus*-related genera should better be merged into one genus; the six* Sorbus*-related genera would be better classified into at least two genera; and all* Photinia*-related genera should be accepted as distinct genera. For a more reliable classification, phylogeny based on the whole chloroplast genomes of representative species from each genus should be used. Such a strategy has been practiced on many seed plants (e.g., [76–83]). Besides, the nuclear genes, especially the single copy nuclear genes in diploid species such as starch-branching enzyme (Sbe; [84]), should be considered with priority to document the origins of Maleae, as the maternally transmitted markers can only tell one aspect of the whole story. Application of nuclear genome information in phylogenetic reconstruction of Maleae also seems feasible, because the genomes of several species in the tribe have been sequenced, and the resequencing of many species is underway. We are expecting a completely resolved phylogeny of Maleae, and that is why we do not provide a formal taxonomic treatment in this study.

## Figures and Tables

**Table 1:** Representative species of Maleae, other major lineages of Amygdaloideae, and an outgroup used in this study, together with sample vouchers and sampling localities.

Taxon	Voucher	Locality
*Amelanchier arborea* (F. Michx.) Fernald	S. L. Zhou BOP022147	Herbarium, Institute of Botany, CAS (PE)
*Aria nivea* Host	S. L. Zhou BOP022174	Herbarium, Institute of Botany, CAS (PE)
*Aronia melanocarpa* (Michx.) Elliott	S. L. Zhou BOP022150	Herbarium, Institute of Botany, CAS (PE)
*Chaenomeles speciosa* (Sweet) Nakai	S. L. Zhou BOP010027	Beijing Botanical Garden, CAS
*Chamaemeles coriacea* Lindl.	K. R. Roberson 4775	Illinois Natural History Survey Herbarium (ILLS)
*Chamaemespilus alpina* (Mill.) K. R. Robertson & J. B. Phipps	K. R. Roberson 5276	Illinois Natural History Survey Herbarium (ILLS)
*Cormus domestica* (L.) Spach	S. L. Zhou BOP022163	Herbarium, Institute of Botany, CAS (PE)
*Cotoneaster multiflorus* Bunge	S. L. Zhou BOP010016	Beijing Botanical Garden, CAS
*Crataegus kansuensis* E. H. Wilson	S. L. Zhou BOP010010	Beijing Botanical Garden, CAS
*Cydonia oblonga* Mill.	S. L. Zhou BOP010020	Beijing Botanical Garden, CAS
*Dichotomanthes tristaniicarpa* Kurz	S. L. Zhou BOP027700	Kunming Botanical Garden, CAS
*Docynia delavayi* (Franch.) C. K. Schneid.	S. L. Zhou BOP027738	Xishuangbanna Tropical Botanical Garden, CAS
*Docyniopsis tschonoskii* (Maxim.) Koidz.	S. L. Zhou BOP022164	Herbarium, Institute of Botany, CAS (PE)
*Eriobotrya japonica* (Thunb.) Lindl.	S. L. Zhou BOP003044	Beijing Botanical Garden, CAS
*Eriolobus kansuensis* (Batalin) C. K. Schneid.	S. L. Zhou BOP011038	Huludao, Liaoning, CHN
*Gillenia trifoliata *(L.) Moench	S. L. Zhou BOP022159	Herbarium, Institute of Botany, CAS (PE)
*Heteromeles arbutifolia* (Dryand.) M. Roem.	S. L. Zhou BOP022160	Herbarium, Institute of Botany, CAS (PE)
*Kageneckia crataegifolia* Lindl.	S. L. Zhou BOP022176	Herbarium, Institute of Botany, CAS (PE)
*Lindleyella schiedeana* (Schltdl.) Rydb.	S. L. Zhou BOP022161	Herbarium, Institute of Botany, CAS (PE)
*Malacomeles denticulata* (Kunth) G. N. Jones	S. L. Zhou BOP022179	Herbarium, Institute of Botany, CAS (PE)
*Malus baccata *(L.) Borkh.	S. L. Zhou BOP016421	Beijing Botanical Garden, CAS
*Mespilus germanica* L.	S. L. Zhou BOP022165	Herbarium, Institute of Botany, CAS (PE)
*Micromeles folgneri *C. K. Schneid.	S. L. Zhou BOP017025	Harbin, Heilongjiang, CHN
*Osteomeles schwerinae *C. K. Schneid.	S. L. Zhou BOP027697	Kunming Botanical Garden, CAS
*Peraphyllum ramosissimum* Nutt. ex Torr. & A. Gray	S. L. Zhou BOP022168	Herbarium, Institute of Botany, CAS (PE)
*Photinia serratifolia* (Desf.) Kalkman	S. L. Zhou BOP027633	Beijing Botanical Garden, CAS
*Pourthiaea arguta* var. *salicifolia* (Decne.) Hook. f.	S. L. Zhou BOP022178	Herbarium, Institute of Botany, CAS (PE)
*Pseudocydonia sinensis *(Dum. Cours.) C. K. Schneid.	S. L. Zhou BOP010349	Beijing Botanical Garden, CAS
*Pyracantha fortuneana* (Maxim.) H. L. Li	S. L. Zhou BOP003043	Beijing Botanical Garden, CAS
*Pyrus bretschneideri* Rehder	S. L. Zhou BOP010065	Beijing Botanical Garden, CAS
*Rhaphiolepis indica* (L.) Lindl.	S. L. Zhou BOP016354	Kunming Botanical Garden, CAS
*Sorbus aucuparia *L.	S. L. Zhou BOP016569	Harbin, Heilongjiang, CHN
*Stranvaesia davidiana* Decne.	S. L. Zhou BOP027698	Kunming Botanical Garden, CAS
*Torminalis clusii* (M.Roem.) K. R. Robertson & J. B. Phipps	K. R. Roberson 5275	Illinois Natural History Survey Herbarium (ILLS)
*Vauquelinia corymbosa* Corrêa ex Humb. & Bonpl.	S. L. Zhou BOP022177	Herbarium, Institute of Botany, CAS (PE)
Other lineages		
*Physocarpus amurensis* (Maxim.) Maxim.	S. L. Zhou BOP010043	Beijing Botanical Garden, CAS
*Prinsepia sinensis* (Oliv.) Oliv. ex Bean	S. L. Zhou BOP010133	Beijing Botanical Garden, CAS
*Rhodotypos scandens* (Thunb.) Makino	S. L. Zhou BOP010022	Beijing Botanical Garden, CAS
*Sorbaria sorbifolia* (L.) A. Braun	S. L. Zhou BOP016568	Beijing Botanical Garden, CAS
*Spiraea pubescens* Turcz.	S. L. Zhou BOP010042	Beijing Botanical Garden, CAS
Outgroup		
*Rosa rugosa *Thunb.	S. L. Zhou BOP010536	Beijing Botanical Garden, CAS

**Table 2:** Primers used to amplify and sequence 15 regions of chloroplast genome of Rosaceae.

Gene regions	Forward primer (5′-3′)	Reverse primer (5′-3′)	Reference
*atpB-rbcL*	TTTCCTAATAATTGCTGTACC	ACAGTTGTCCATGTACCAGTAG	Dong et al., 2013 [45]
*matK*	TCTAGCACACGAAAGTCGAAGT	CGATCTATTCATTCAATATTTC	Dong et al., 2013 [45]
*ndhF*	ACACCAACGCCATTCGTAATGCCATC	AAGATGAAATTCTTAATGATAGTTGG	Dong et al., 2013 [45]
*petA-psbJ*	CTAATGTGGGTGGATTTGGTCA	ATGGCCGATACTACTGGAAGG	This study
*psbA-trnH*	GAACCCGCGCATGGTGGATTCAC	TGGCTCCCTATTCAGTGCTATGC	This study
*psbM-trnD*	GTTTTTACGTATATTATAAGTA	GTTCAAATCCAGCTCGGCCCA	This study
*rbcL*	ATGTCACCACAAACAGAGACTAAAGC	TTCCATACTTCACAAGCAGCAGCTAG	Dong et al., 2013 [45]
*rpl16*	TCCCGAAATAATGAATTGAGTTCG	TCAGAGAAGGTAGGGTTCCCCT	Dong et al., 2013 [45]
*rpl20-rps12*	ATGATCTCATTGGAAATCATATAAAG	AGGGTAATGATCCATCAACCGGC	Dong et al., 2013 [45]
*rps16*	GTGGTAGAAAGCAACGTGCGACTT	TCGGGATCGAACATCAATTGCAAC	Oxelman et al., 1997 [85]
*trnC-ycf6*	GGCGGCATGGCCGAGTGGTAAGGC	TCCACTTCTTCCCCATACTACGA	Dong et al., 2013 [45]
*trnH-rpl2*	AGCCAACACTTAGATCCGGCTCTAC	GATTTGTGAATCCACCATGCGCG	Dong et al., 2013 [45]
*trnL-trnF*	GTTCAAGTCCCTCTATCCCCA	GATTTTCAAGAACGGGAATCTTA	Dong et al., 2013 [45]
*trnS-trnG*	AAACTCTTCGTTTACACAGTAGTGA	CTTTTACCACTAAACTATACCCGC	Dong et al., 2013 [45]
*ycf1*	TCTCGACGAAAATCAGATTGTTGTGAAT	ATACATGTCAAAGTGATGGAAAA	Dong et al., 2015 [46]

**Table 3:** Variability of 15 chloroplast regions among species in Maleae and the maximum parsimony tree scores of all taxa.

Gene regions	*N* ^*∗*^	La	Sc	*S*	Is	NH	*π*	*k*	*L* ^*∗*^	CI^*∗*^	RI^*∗*^
*atpB-rbcL*	39	796	659	33	9	25	0.00422	4.15	177	0.921	0.781
*matK*	41	2045	1761	167	47	32	0.00879	8.17	663	0.741	0.538
*ndhF*	39	1973	1821	148	38	29	0.00937	7.5	694	0.745	0.567
*petA-psbJ*	34	1370	1072	155	36	27	0.01046	11.31	554	0.791	0.629
*psbA-trnH*	37	283	222	34	10	15	0.02547	12.01	196	0.789	0.436
*psbM-trnD*	34	1503	1225	126	33	27	0.00901	8.38	589	0.81	0.666
*rbcL*	38	1353	1293	60	25	25	0.00539	4.43	296	0.547	0.442
*rpl16*	40	1178	940	124	31	32	0.00804	10.53	463	0.689	0.518
*rpl20-rps12*	37	743	677	42	15	30	0.00710	5.65	230	0.752	0.617
*rps16*	39	964	772	84	22	28	0.00999	8.71	378	0.746	0.639
*trnC-ycf6*	36	1032	810	90	30	28	0.01084	8.72	525	0.785	0.616
*trnH-rpl2*	36	205	189	11	5	5	0.00391	5.37	196	0.789	0.436
*trnL-trnF*	41	1088	944	90	24	26	0.00783	8.27	420	0.757	0.612
*trnS-trnG*	36	895	661	102	20	27	0.01011	11.4	470	0.781	0.528
*ycf1*	34	866	742	88	21	28	0.01210	10.16	489	0.765	0.595

*N*, number of species; La, aligned length; Sc, sites considered; *S*, number of polymorphic sites, excluding sites with missing data; Is, parsimony-informative site; NH, number of haplotypes; *π*, nucleotide diversity; *k*, average number of nucleotide differences; *L*, the tree length; CI, consistency index; RI, retention index.  ^*∗*^Including all taxa.

## Supplementary Materials

Supplementary Material Supplementary Table S1: sequences downloaded from GenBank in this study with accession numbers. Supplementary Table S2: newly generated sequences with GenBank accession numbers in this study.
