# Supplementary material for: Phylogeny of Maleae (Rosaceae) Based on Multiple Chloroplast Regions: Implications to Genera Circumscription
Source: Biomed Res Int. 2018 Mar 19;2018:7627191. doi: 10.1155/2018/7627191 (PMC5884435; doi:10.1155/2018/7627191)
Supplement: Supplementary Material — Supplementary Table S1: sequences downloaded from GenBank in this study with accession numbers. Supplementary Table S2: newly generated sequences with GenBank accession numbers in this study. [file 7627191.f1.pdf]

Supplementary Table S1. Sequences downloaded from GenBank in this study with public database accession numbers.

Taxon	*atpB-rbcL*	*matK*	*ndhF*	*rpl16*	*rps16*	*trnL-trnF*
Chamaemeles coriacea Lindl.	-	DQ860454	DQ851507	DQ860423	DQ848689	DQ863226
Chamaemespilus alpina (Mill.) K. R. Robertson & J. B. Phipps	DQ860485	DQ860455	DQ851508	DQ860424	DQ848690	DQ863227
Cormus domestica (L.) Spach	DQ860486	DQ860456	DQ851509	DQ860425	DQ848691	DQ863228
Heteromeles arbutifolia (Dryand.) M. Roem.	-	-	-	-	DQ848699	-
Malacomeles denticulata (Kunth) G. N. Jones	DQ860495	DQ860465	DQ851530	DQ860434	DQ848700	DQ863237
Torminalis clusii (M.Roem.) K. R. Robertson & J. B. Phipps	DQ860506	DQ860477	DQ851559	DQ851559	DQ848712	DQ863249

Supplementary Table S2. Newly generated sequences with GenBank accession numbers in this study.

Taxon	*petA-psbJ*	*rbcL*	*rpl16*	*rpl20-rps2*	*rps16*	*trnC-ycf6*	*trnD-psbM*	*trnH-psbA*	*trnH-rpl2*	*trnL-F*	*trnS-G*	*ycf1*	*atpB-rbcL*	*matK*	*ndhF*
Sorbaria sorbifolia (L.) A.Braun	MG703578	MG703612	MG703649	MG703684	MG703721	MG703754	MG703790	MG703824	MG703860	MG703896	MG703932	MG703968	MG704002	MG704037	MG704073
Prinsepia sinensis (Oliv.) Oliv. ex Bean	MG703579	MG703613	MG703650	MG703685	MG703722	MG703755	MG703791	MG703825	MG703861	MG703897	MG703933	MG703969	MG704003	MG704038	MG704074
Rhodotypos scandens (Thunb.) Makino	MG703580	MG703614	MG703651	MG703686	MG703723	MG703756	MG703792	MG703826	MG703862	MG703898	MG703934	MG703970	MG704004	MG704039	MG704075
Physocarpus amurensis (Maxim.) Maxim.	MG703581	MG703615	MG703652	MG703687	MG703724	MG703757	MG703793	MG703827	MG703863	MG703899	MG703935	MG703971	MG704005	MG704040	MG704076
Spiraea pubescens Turcz.	MG703582	MG703616	MG703653	MG703688	MG703725	MG703758	MG703794	MG703828	MG703864	MG703900	MG703936	MG703972	MG704006	MG704041	MG704077
Rosa rugosa Thunb.	MG703583	MG703617	MG703654	MG703689	MG703726	MG703759	MG703795	MG703829	MG703865	MG703901	MG703937	MG703973	MG704007	MG704042	MG704078
Amelanchier arborea (F. Michx.) Fernald	MG703584	MG703618	MG703655	MG703690	MG703727	MG703760	MG703796	MG703830	MG703866	MG703902	MG703938	MG703974	MG704008	MG704043	MG704079
Aria nivea Host	-	MG703619	-	-	-	-	-	MG703831	-	MG703903	-	-	-	MG704044	-
Aronia melanocarpa (Michx.) Elliott	MG703585	MG703620	MG703656	MG703691	MG703728	MG703761	MG703797	MG703832	MG703867	MG703904	MG703939	MG703975	MG704009	MG704048	MG704080
Chaenomeles speciosa (Sweet) Nakai	MG703586	MG703621	MG703657	MG703692	MG703729	MG703762	MG703798	MG703833	MG703868	MG703905	MG703940	MG703976	MG704010	MG704049	MG704081
Cormus domestica (L.) Spach	MG703587	MG703622	-	MG703693	-	MG703763	MG703799	MG703834	MG703869	-	MG703941	MG703977	-	-	-
Cotoneaster multiflorus Bunge	MG703588	MG703623	MG703658	MG703694	MG703730	MG703764	MG703800	MG703835	MG703870	MG703906	MG703942	MG703978	MG704011	MG704050	MG704082
Crataegus kansuensis E. H. Wilson	MG703589	MG703624	MG703659	MG703695	MG703731	MG703765	MG703801	MG703836	MG703871	MG703907	MG703943	MG703979	MG704012	MG704051	MG704083
Cydonia oblonga Mill.	MG703590	MG703625	MG703660	MG703696	MG703732	MG703766	MG703802	MG703837	MG703872	MG703908	MG703944	MG703980	MG704013	MG704052	MG704084
Dichotomanthes tristaniicarpa Kurz	MG703591	MG703626	MG703661	MG703697	MG703733	MG703767	MG703803	MG703838	MG703873	MG703909	MG703945	MG703981	MG704014	MG704053	MG704085
Docynia delavayi (Franch.) C. K. Schneid.	MG703592	MG703627	MG703662	MG703698	MG703734	MG703768	MG703804	MG703839	MG703874	MG703910	MG703946	MG703982	MG704015	MG704054	MG704086
Docyniopsis tschonoskii (Maxim.) Koidz.	MG703593	MG703628	MG703663	MG703699	MG703735	MG703769	MG703805	MG703840	MG703875	MG703911	MG703947	MG703983	MG704016	MG704055	MG704087
Eriobotrya japonica (Thunb.) Lindl.	MG703594	MG703629	MG703664	MG703700	MG703736	MG703770	MG703806	MG703841	MG703876	MG703912	MG703948	MG703984	MG704017	MG704056	MG704088
Eriolobus kansuensis (Batalin) C. K. Schneid.	-	-	MG703665	MG703701	-	MG703771	-	MG703842	-	MG703913	MG703949	-	MG704018	MG704047	-
Gillenia trifoliata (L.) Moench	MG703595	MG703630	MG703666	MG703702	MG703737	MG703772	MG703807	MG703843	MG703877	MG703914	MG703950	MG703985	MG704019	MG704057	MG704089
Heteromeles arbutifolia (Dryand.) M. Roem.	-	MG703631	MG703667	MG703703	-	MG703773	-	-	MG703878	MG703915	MG703951	-	MG704020	MG704045	MG704090
Kageneckia crataegifolia Lindl.	MG703596	MG703632	MG703668	MG703704	MG703738	MG703774	MG703808	MG703844	MG703879	MG703916	MG703952	MG703986	MG704021	MG704058	MG704091
Lindleyella schiedeana (Schltdl.) Rydb.	-	MG703633	MG703669	MG703705	MG703739	-	-	-	MG703880	MG703917	-	-	MG704022	MG704046	MG704092
Malacomeles denticulata (Kunth) G. N. Jones	MG703597	MG703634	-	MG703706	-	MG703775	MG703809	MG703845	MG703881	-	MG703953	MG703987	-	-	-
Malus baccata (L.) Borkh.	MG703598	MG703635	MG703670	MG703707	MG703740	MG703776	MG703810	MG703846	MG703882	MG703918	MG703954	MG703988	MG704023	MG704059	MG704093
Mespilus germanica L.	MG703599	MG703636	MG703671	MG703708	MG703741	MG703777	MG703811	MG703847	MG703883	MG703919	MG703955	MG703989	MG704024	MG704060	MG704094
Micromeles folgneri C. K. Schneid.	MG703600	MG703637	MG703672	MG703709	MG703742	MG703778	MG703812	MG703848	MG703884	MG703920	MG703956	MG703990	MG704025	MG704061	MG704095
Osteomeles schwerinae C. K. Schneid.	MG703601	MG703638	MG703673	MG703710	MG703743	MG703779	MG703813	MG703849	MG703885	MG703921	MG703957	MG703991	MG704026	MG704062	MG704096
Peraphyllum ramosissimum Nutt. ex Torr. & A. Gray	MG703602	MG703639	MG703674	MG703711	MG703744	MG703780	MG703814	MG703850	MG703886	MG703922	MG703958	MG703992	MG704027	MG704063	MG704097
Photinia serratifolia (Desf.) Kalkman	MG703603	MG703640	MG703675	MG703712	MG703745	MG703781	MG703815	MG703851	MG703887	MG703923	MG703959	MG703993	MG704028	MG704064	MG704098
Pourthiaea arguta var. *salicifolia* (Decne.) Hook. f.	MG703604	MG703641	MG703676	MG703713	MG703746	MG703782	MG703816	MG703852	MG703888	MG703924	MG703960	MG703994	MG704029	MG704065	MG704099
Pseudocydonia sinensis (Dum.Cours.) C. K. Schneid.	MG703605	MG703642	MG703677	MG703714	MG703747	MG703783	MG703817	MG703853	MG703889	MG703925	MG703961	MG703995	MG704030	MG704066	MG704100
Pyracantha fortuneana (Maxim.) H. L. Li	MG703606	MG703643	MG703678	MG703715	MG703748	MG703784	MG703818	MG703854	MG703890	MG703926	MG703962	MG703996	MG704031	MG704067	MG704101
Pyrus bretschneideri Rehder	MG703607	MG703644	MG703679	MG703716	MG703749	MG703785	MG703819	MG703855	MG703891	MG703927	MG703963	MG703997	MG704032	MG704068	MG704102
Rhaphiolepis indica (L.) Lindl.	MG703608	MG703645	MG703680	MG703717	MG703750	MG703786	MG703820	MG703856	MG703892	MG703928	MG703964	MG703998	MG704033	MG704069	MG704103
Sorbus aucuparia L.	MG703609	MG703646	MG703681	MG703718	MG703751	MG703787	MG703821	MG703857	MG703893	MG703929	MG703965	MG703999	MG704034	MG704070	MG704104
Stranvaesia davidiana Decne.	MG703610	MG703647	MG703682	MG703719	MG703752	MG703788	MG703822	MG703858	MG703894	MG703930	MG703966	MG704000	MG704035	MG704071	MG704105
Vauquelinia corymbosa Corrêa ex Humb. & Bonpl.	MG703611	MG703648	MG703683	MG703720	MG703753	MG703789	MG703823	MG703859	MG703895	MG703931	MG703967	MG704001	MG704036	MG704072	MG704106
